# The Redcap Clinic: Exploring CAMHS and Paediatric Staff Attitudes to a Newly Launched Physical Health Service for Young People With Eating Disorders

**DOI:** 10.1192/bjo.2026.11645

**Published:** 2026-06-30

**Authors:** Harriet Greenstone, Chloe Harrison, Monica Potter

**Affiliations:** Avon and Wiltshire Mental Health Partnership Trust, Bristol, United Kingdom

## Abstract

**Aims:**

This project evaluated a new service for CAMHS patients with Eating Disorders within Avon and Wiltshire Mental Health Partnership Trust. The service (‘the Redcap clinic’) was commissioned in 2024, and began operating in January 2025, providing physical health review (physical observations, bloods, ECGs) for ED patients under 18. The clinic is run at the Bristol Royal Hospital for Children by paediatricians specialising in Eating Disorders, and interpreted results are shared with locality CAMHS teams. The key aim of the clinic is to safely and effectively assess the physical health needs of this patient cohort, facilitating collaborative clinical decision-making between paediatrics and psychiatry. This project aimed to explore clinicians’ views of the new service.

**Methods:**

A nine-question electronic survey was disseminated amongst CAMHS and paediatric staff referring to, or running, the clinic. The survey ran over 90 days (September – November 2025), using convenience sampling. Mixed methods were used to interpret the results; the first five questions were analysed using descriptive quantitative methods. The latter four questions elicited free-text responses, on which the lead researcher conducted a thematic analysis based on Braun and Clarke’s method of thematic analysis.

**Results:**

In total, 24 responses were received from members across the MDT in both CAMHS and Paediatrics. 100% of respondents felt that the clinic provided joined-up care, and 95% of respondents felt that the clinic allowed faster access to investigations. Views of respondents indicated that the clinic: 1) provided joined-up care between paediatrics and psychiatry; 2) allowed patients faster access to tests compared to appointments in primarycare; 3) was convenient for patients compared to having tests at the GP; 4) saved clinicians’ time; 5) had a robust and effective system for results feedback. Qualitative analysis of free-text responses generated five themes of the clinic’s impact on professionals – ‘saving time in community CAMHS’, ‘meeting patient needs’, ‘confidence in the system’, ‘facilitating safer decision-making’, and ‘connection with experts’. Regarding the impact of the clinic on patients and families, qualitative analysis of responses yielded four themes – ‘accessibility’, ‘containment’, ‘appropriate/adapted’ and ‘continuity’.

**Conclusion:**

Overall, clinicians’ feedback on the Redcap clinic was positive; the clinic was felt to facilitate safe, effective, and joined-up care for a vulnerable group of young people with specific physical and psychological health needs. These results have supported local efforts for ongoing commissioning. Suggestions for further service development are discussed, as are future plans to explore feedback from young people and families using the service.

